# Dietary Patterns in Italy and the Risk of Renal Cell Carcinoma

**DOI:** 10.3390/nu12010134

**Published:** 2020-01-02

**Authors:** Michela Dalmartello, Francesca Bravi, Diego Serraino, Anna Crispo, Monica Ferraroni, Carlo La Vecchia, Valeria Edefonti

**Affiliations:** 1 Department of Clinical Sciences and Community Health, Università degli Studi di Milano, via A. Vanzetti, 5, 20133 Milan, Italy; michela.dalmartello@unimi.it (M.D.); francesca.bravi@unimi.it (F.B.); monica.ferraroni@unimi.it (M.F.); carlo.lavecchia@unimi.it (C.L.V.); 2 Cancer Epidemiology Unit, Centro di Riferimento Oncologico di Aviano (CRO) IRCCS, via F. Gallini, 2, 33080 Aviano, Italy; serrainod@cro.it; 3 Epidemiology and Biostatistics Unit, Istituto Nazionale Tumori—IRCCS “Fondazione G. Pascale”, via M. Semmola, 80131 Naples, Italy; a.crispo@istitutotumori.na.it

**Keywords:** kidney cancer, renal cell carcinoma, case-control study, dietary patterns, diet, factor analysis

## Abstract

Background: Conclusive evidence on foods, nutrients, or dietary patterns and the risk of renal cell carcinoma (RCC) is lacking in the literature. Methods: We considered data from an Italian hospital-based case–control study (1992–2004) on 767 incident RCC cases and 1534 controls. *A posteriori* dietary patterns were identified by applying principal component factor analysis on 28 nutrients derived from a 78-item food-frequency questionnaire. We estimated the odds ratios (ORs) of RCC and corresponding 95% confidence intervals (CIs) for each quartile category (compared to the lowest one) using conditional multiple logistic regression models providing adjustment for major confounding factors. Results: We identified four dietary patterns, named “Animal products”, “Starch-rich”, “Vitamins and fiber”, and “Cooking oils and dressings”. Higher intakes of the “Starch-rich” pattern were positively associated with RCC risk (OR = 1.38, 95% CI: 1.04–1.82 for the highest quartile, *p* = 0.018). The association was inverse with the “Cooking oils and dressings” pattern (OR = 0.61, 95% CI: 0.47–0.80, *p* < 0.001), whereas no association was found with “Animal products” and “Vitamins and fiber” patterns. Conclusions: Higher intakes of starch-related foods may increase RCC risk, whereas consumption of olive and seed oils may favorably influence RCC risk.

## 1. Introduction

Kidney cancer accounts for about 2% of all cancers worldwide, with more than 400,000 new cases identified every year. In Europe, it is on average the 8th most common cancer [1]. Approximately 70% of kidney cancer cases among adults are clear cell renal cell carcinomas (RCCs) [2].

Temporal and geographic variations in incidence rates characterize kidney cancer. Intra-country regional differences have been reported in Italy, with higher incidence rates in the Northern areas [3]. Incidence rates have been also reported to increase in the world since the 70s, with an average annual percentage increase between 2% and 3% [4]. Modifications of lifestyle and exposures to risk factors, aa well as in tumor detection and diagnostic procedures over time, are hypothesized to account for these temporal trends [5]. Furthermore, kidney cancer is one of the few neoplasms with an unfavorable trend in mortality over recent decades [6].

Apart from genetic predisposition, established risk factors for kidney cancer are tobacco smoking, overweight, and history of chronic kidney diseases and hypertension; typically, alcohol drinkers have a 20% reduction in risk, compared with light- and non-drinkers [7,8]. Conclusive evidence on the role of diet on kidney cancer risk is still lacking. Indeed, prospective cohort studies showed mostly null or nonsignificant associations with fruit and vegetable consumption, or a modest reduction in risk for the highest intakes of these foods; the evidence is also scanty for nutrients, with null associations reported by most studies that examined protein or fat intakes [7].

When the effect of a single nutrient is too limited to be observed, the overall effect of multiple nutrients combined in a dietary pattern (DP) may be sufficiently large to be detectable; in addition, DPs may address the issues of collinearity among nutrients and interdependencies between nutrients and foods [9]. However, only a few studies have considered the association between *a priori* [10] and *a posteriori* [11,12,13] DPs and kidney cancer, including RCC, with null or modest associations.

To further investigate this topic, we carried out an exploratory principal component factor analysis (PCFA) to identify *a posteriori* dietary patterns, based on an Italian case-control study on RCC.

## 2. Materials and Methods

### 2.1. Design and Participants

A multicentric case-control study on RCC was undertaken between 1992 and 2004 in the following Italian areas: the greater Milan area, and the provinces of Pordenone and Udine, in northern Italy, the province of Latina in central Italy, and the urban area of Naples in southern Italy [14]. Cases were 767 patients [273 women (35.6%) and 494 men (64.4%), younger than 80 years old (median age: 62, range: 24–79 years)] with incident, histologically confirmed RCC [International Classification of Diseases (ICD)-9 189.0], admitted to major teaching and general hospitals of the study areas. Cancers of the renal pelvis and ureter (ICD-9, 189.1–189.2) were excluded. According to the 2004 World Health Organization Classification [15], 54% of RCC were clear cell, 7% papillary, 1% chromophobe, 12% unclassified, and 26% unknown. Controls were 1534 subjects [546 women (35.6%) and 988 men (64.4%), younger than 80 years old (median age: 62, range 22–79 years)] admitted to the same hospitals for various acute non-neoplastic diseases, with no relationship with known risk factors for RCC. Cases and controls were matched by study center, sex and quinquennia of age, with a ratio of 1:2. Twenty-six percent of controls had an admission diagnosis of traumas (mostly fractures and sprains), 32% of other orthopedic conditions (e.g., disc disorders and low back pain), 14% of surgical conditions and 27% of various other acute conditions, such as nose, ear, eye, skin, or dental conditions. Less than 5% of both cases and controls approached did not agree to be included in the study. The study was undertaken according to the Declaration of Helsinki, and the protocol was approved by the Local Ethics Committees in force at the time of data collection.

All subjects gave their written informed consent for inclusion before they participated in the study. Centrally-trained interviewers collected information from both cases and controls during their hospital stay using the same structured questionnaire, concerning sociodemographic characteristics, anthropometric measures and lifestyle factors, personal medical history, and family history of cancer among first-degree relatives.

Moreover, the interviewers administered to the participants a food frequency questionnaire (FFQ) concerning their usual diet during the 2 years before cancer diagnosis (or hospital admission for controls). The FFQ included 78 foods and beverages belonging to the following 6 groups: (1) first courses (including bread and cereals); (2) second courses (including meat, fish, and cheese); (3) vegetables; (4) fruits; (5) sweets, cakes, and soft drinks, and (6) milk, coffee, tea, and sweeteners. Additional questions investigated consumption of alcoholic beverages. Participants were asked to report for each dietary item the usual weekly frequency of consumption. For selected vegetables and fruits, which are supposed to be consumed only in a specific season, the duration of consumption in months was asked in addition to the weekly consumption; occasional consumptions, defined as less than once a week but at least once per month, were coded as 0.5 per week. We estimated selected nutrient intakes as well as total energy intake using an Italian food composition database [16,17]. Validity [18] and reproducibility [19] of the FFQ were tested using two 7-day dietary records.

### 2.2. Statistical Analysis

#### 2.2.1. Factorability of the Original Matrix

We carried out the analyses on a comprehensive list of 28 nutrients, including minerals, micro- and macro-nutrients, which are representative of the traditional Italian diet and are likely related to cancer risk. We evaluated whether the correlation matrix of the original nutrients (based on all participants) was factorable, by visual inspection of the matrix as well as statistical procedures, including Bartlett’s test of sphericity, overall (Kaiser–Meyer–Olkin) and individual measures of sampling adequacy [20]. Since reassuring results were obtained (Table 1), we applied a factor analysis to derive the *a posteriori* DPs.

#### 2.2.2. Dietary Pattern Identification

Exploratory PCFA [21] was carried out on the previously assessed correlation matrix to provide a description of the variance–covariance structure among nutrients in terms of a smaller number of underlying unobservable and randomly varying factors that are usually interpreted as DPs. We selected the number of factors to retain according to the following criteria: factor eigenvalue greater than 1, scree-plot examination, and factors interpretability [21]. We used a varimax rotation to obtain a simpler loading structure. The name of each factor was defined taking into account nutrients with rotated factor loadings in absolute value ≥ 0.63. These are named ‘dominant nutrients’ hereafter. Indeed, choosing a 0.63 cut-off implies a minimum contribution of any factor to any nutrient’s total variance of 0.63^2^~0.40 [22].

Factor scores, calculated for each subject and each pattern, indicate the degree to which each participant’s diet is consistent with the identified pattern. We calculated factor scores using the weighted least squares method [23].

#### 2.2.3. Reproducibility, Reliability, and Validity of the Identified Dietary Patterns

To confirm internal reproducibility of the identified DPs, we carried out additional analyses choosing a different estimation procedure (i.e., principal axis factor analysis) and a different estimation method for deriving factor scores (i.e., multiple regression method) [21]. In addition, we split the original dataset in 2 randomly selected subsets, and carried out separate factor analyses according to the same approach of the main analysis (split-half approach). Cases and controls were equally divided in the 2 groups. The procedure was repeated several times with different seeds for random assignment of subjects [23].

Given the reassuring results from these checks (correlation coefficients between different sets of factors scores equal to 0.99 and congruence coefficients between factor loadings from similar DPs >0.90), we carried out all the subsequent analyses on the factor scores derived from the overall PCFA with varimax rotation using the weighted least squares method.

To evaluate factor reliability and potentially improve the identified DPs, we assessed the internal consistency of nutrients with a factor loading greater or equal to 0.40 in absolute value on any factor through the standardized Cronbach’s coefficient *alpha* [20]. In detail, for each factor we computed one coefficient *alpha* and several coefficient *alphas when-item-deleted,* which assessed the importance of each nutrient within the corresponding DP.

To improve interpretability of the identified DPs, we also calculated the Spearman rank correlation coefficients between the continuous factor scores obtained from PCFA and the weekly intake of 29 selected food groups, defined on the same data and derived from the original 78 food items on the basis of similarities in content and use [23].

#### 2.2.4. Risk Estimates

For each factor, we categorized all the participants into 4 groups based on the quartile distribution of each factor score among the controls. We estimated the odds ratios (ORs) of RCC, as well as their 95% confidence intervals (CIs), for each quartile category compared to the lowest one (reference category), fitting multiple logistic regression models [24], conditioned on study center, sex and quinquennia of age and adjusted for years of education (<7, 7–11, >11 years), period of interview (≤1998, >1998), family history of kidney cancer in first-degree relatives (no, yes), hypertension (no, yes), tobacco smoking (never smoker, former smoker, current smoker <20 cigarettes/day, current smoker ≥20 cigarettes/day), alcohol drinking (never drinker, former drinker, current drinker <21 drinks/week, current drinker ≥21 drinks/week), and body mass index (BMI, <25, 25–30, >30 kg/m^2^). We fitted one model for each DP, including matching variables and confounding factors too. Moreover, after varimax rotation, we were allowed to fit an overall model including all the DPs (quartile categories) together, as well as matching variables and confounding factors, in order to obtain an estimate of the effect of each DP that accounted for the role of the remaining DPs. Tests for linear trend for all these models were computed using the medians calculated within category among the controls. Stratified analyses were performed by education, history of hypertension, BMI, tobacco smoking, and alcohol drinking. The presence of heterogeneity across strata was assessed using the likelihood ratio test comparing models with and without the interaction term between stratification variable and DP under consideration.

We carried out the analyses using SAS software, version 9.4 (SAS Institute, Inc., Cary, North Carolina, NC, USA).

## 3. Results

The correlation matrix of the original nutrients was adequate for factor analysis. The Bartlett’s test of sphericity was statistically significant (*p* < 0.001), allowing us to reject the null hypothesis that the correlation matrix is the identity matrix. The overall measure of sampling adequacy was equal to 0.84, thus reassuring that the sample size was large enough, as compared to the number of nutrients under consideration. Moreover, the individual measures of sampling adequacy were satisfactory, with 6 nutrients having measures ≥0.90, 14 having measures in the 0.80s, 5 in the 0.70s, and only 3 nutrients with measures below 0.70.

Table 2 gives the factor loading matrix for the 4 retained DPs, the corresponding communality estimates, and the proportion of explained variance. The retained DPs explained 74.5% of the total variance in the original dataset. The first pattern, labeled “Animal products”, had the highest factor loadings (which means the higher contribution from) on calcium, animal protein, riboflavin, phosphorus, cholesterol, saturated fatty acids, and zinc. The second pattern, named “Starch-rich” had the highest factor loadings on starch, vegetable protein, and sodium. The third pattern, labeled “Vitamins and fiber”, was characterized by the greatest factor loadings on vitamin C, total fiber, soluble carbohydrates, beta-carotene equivalents, potassium, and total folate. The fourth pattern, labeled “Cooking oils and dressings”, had the greatest factor loadings on vitamin E, linoleic acid, and linolenic acid. All the nutrients had at least one factor loading >0.30, thus confirming an important role of all the selected nutrients in this analysis. The proportion of nutrient variance explained by all the retained factors (i.e., the communality estimates) were generally satisfactory and were greater than 0.70 for most nutrients.

Standardized Cronbach’s coefficient *alphas* had high values (at least 0.94) for all the factors and the majority of the standardized coefficient *alphas when-item-deleted* were smaller than the corresponding coefficient *alpha* for the same factor, which indicates an adequate internal consistency of the nutrients on the identified factors (data not shown).

Table 3 shows the Spearman rank correlation coefficients between the PCFA-based scores and the intake of 29 food groups and dressings. These correlations allowed us to confirm the interpretation and labeling of the nutrient-based DPs, which we provided on the basis of factor scores. Indeed, for the “Animal products” DP, the greatest values were with cheese, milk, eggs, desserts, liver, and red meat; for the “Starch-rich” DP, the greatest values of the Spearman coefficient were with bread and red meat; for the “Vitamins and fiber” DP, the greatest values were with fruits and vegetables, including other fruits, citrus fruits, fruiting vegetables, other vegetables, root vegetables, leafy vegetables, pulses, and milk; for the “Cooking oils and dressings” DP, the greatest values of the Spearman coefficient were with unspecified seed oils, red meat, and olive oil.

Table 4 provides the distribution of subjects’ characteristics at baseline according to DP quartile categories. Moderate-to-high alcohol drinking was positively associated to the “Animal products”, “Starch-rich”, and “Cooking oils and dressings” DPs (*p* < 0.0001). Current tobacco smoking was positively associated to the “Starch-rich” DP (*p* < 0.0001) and inversely associated to the “Vitamins and fiber” DP (*p* = 0.003). In addition, hypertension was inversely related to the “Starch-rich” (*p* = 0.003) and “Vitamins and fiber” DPs (*p* = 0.030), whereas a history of kidney cancer in first-degree relatives was inversely related to the “Animal products” DP (*p* = 0.014). BMI was positively related to the “Starch-rich” (*p* = 0.040) and “Cooking oils and dressings” (*p* < 0.0001) DPs. Energy intake was positively related to all DPs (*p* < 0.0001).

Table 5 shows the ORs of RCC, as well as the corresponding 95% CIs, in quartiles of factor scores of the four identified DPs. Results are obtained from the overall model including all the 4 patterns together, after adjustment for confounding factors. Higher intakes of the dominant nutrients for the “Starch-rich” DP were positively associated with RCC risk (OR = 1.38, 95% CI: 1.04–1.82 for the highest vs the lowest score quartile, *p* = 0.018). An inverse association was found between RCC and the “Cooking oils and dressings” DP (OR = 0.61, 95% CI: 0.47–0.80, *p* < 0.001), whereas no association was observed for the “Animal products” (OR = 0.87, 95% CI: 0.67–1.14) or the “Vitamins and fiber” (OR = 1.02, 95% CI: 0.79–1.32) DPs. The estimates obtained fitting separate models were largely consistent with those of the overall model.

Table 6 and Table 7 give the ORs of RCC and corresponding CIs for the identified DPs in strata of education, history of hypertension, BMI, tobacco smoking, and alcohol drinking. No material heterogeneity was present across strata for the “Starch-rich” and “Cooking oils and dressings” DPs; the point estimates for the last quartile category of the two DPs were similar to the overall estimate, although the CIs included unity in some cases. Heterogeneity across strata was detected for the “Animal Products” DP in strata of smoking and for the “Vitamins and fiber” DP in strata of BMI and smoking; however, most of the CIs in those strata included unity.

## 4. Discussion

The present analysis allowed us to identify four main DPs that explained ~75% of the total variance in the nutrient intakes of our Italian population. After mutual adjustment for all the other DPs, the “Starch-rich” DP was associated with an excess in RCC risk, whereas the “Cooking oils and dressings” DP showed a protective effect. In addition, the validity of the identified DPs, as explored in terms of related food groups, lifestyle and medical characteristics, was reassuring.

Current evidence on the role of diet on kidney cancer risk is mostly based on single foods, food groups or nutrients and it is still limited and inconclusive [7]. Only three previous studies [11,12,13] have considered the role of food-based DPs on kidney cancer or RCC risk, while no study have examined nutrient-based DPs. Therefore, comparison between our results and the previous ones is only tentative. In addition, any comparison of DPs across different studies depends on the set of food groups (or nutrients) originally selected for the analysis within each study.

Based on 93 RCC cases from 46,562 women in 14 years of follow-up, a study from the Swedish Mammography Cohort [13] identified a “Healthy”, “Western”, and “Drinker” DP, which accounted for 25% of total explained variance. The authors reported also the correlation coefficients between the food-based DPs and nutrient intakes, thus facilitating the comparison between their food-based and our nutrient-based DPs. The “Healthy” DP was strongly correlated with vitamin C, beta-carotene, and total fiber and it is hence similar to our “Vitamins and Fiber” DP; both DPs showed a nonsignificant association with RCC risk. In addition, the “Western” DP identified in that study—which was strongly correlated with total fat, protein, and carbohydrates—shares some elements with our “Animal products” DP and it was also not associated with RCC risk. Finally, their “Drinker” DP weakly protected against RCC risk. Compared to our analysis, they did not consider oils among food groups and had a raw division of carbohydrate-rich foods; however, they did consider alcoholic beverages. This differences in the original dietary items can explain why the Swedish Mammography Cohort study derived a “Drinker” DP and we were able to identify a “Cooking oils and dressings” and a “Starch-rich” DPs.

In a population-based case-control study from Ontario on 461 cases, two similar control-based and sex-specific sets of eight DPs were identified from factor analysis on 69 food items (sex-specific cumulative proportion of variance explained: about 15%) [11]. After model selection on the set of DPs to be included in the final logistic regression, the “Desserts” [based on chocolate, cookies, ice-cream, doughnuts, and potato chips (for all subjects) and also cakes (in females only)] DP was positively associated with RCC (OR = 3.7, 95% CI: 2.0–6.9, for the highest versus lowest quartile) in males only; however, CIs included unity for the “Beef” (based on beef, pork, and lamb) and “Juices“ (based on frozen, powdered, and fresh juices) DPs in males and, in females, for the “Desserts” and the “Unhealthy” DP (based on white, as opposed to dark bread, and peanut butter). The “Beef” DP showed some similarities with the “Animal products” DP in the consumption of meat; however, our “Animal products” DP was also strongly based on dairy products, including milk, cheese, and eggs, and on some desserts. In addition, our “Starch-rich” DP could be considered similar to the “Unhealthy” DP in terms of bread consumption, and therefore of the related RCC risk; however, pasta, rice, and cooked cereals loaded high on DPs originally identified but not selected for the final model. Oils were not included among seasonings queried in the FFQ, so there was no possibility to identify a pattern similar to our “Cooking oils and dressings”.

In a multi-site case-control study from Uruguay [12], factor analysis allowed to identify control-based, sex-specific sets of DPs, including a “Prudent”, a “Traditional”, a “Western”, and a “Drinker” DPs (about 40% of the total variance explained). Although the associations of the identified DPs with kidney cancer went in the expected direction and were similar to ours and to those of other studies [11,13], cases were only 114 and the analysis did not have enough power to reach any firm conclusion.

Among the *a priori* DPs, the European Prospective Investigation into Cancer and Nutrition (EPIC) reported that compliance to the recommendations of the World Cancer Research Fund/American Institute for Cancer Research has a protective effect on kidney cancer [HR = 0.91, 95% CI: 0.85–0.99, for a 1-point increment in the score; HR = 0.71, 95% CI: 0.54–0.93, *p* = 0.03, for the highest category (score range: 5–7 among women and 4–6 among men)] [10]. Diet may, therefore, have an influence on kidney cancer risk when combined with body fatness and physical activity. However, the limited sample size did not allow to assess the effect of single score components on kidney cancer.

The Italian population still offers special opportunities to assess the influence on cancer of high intakes of unsaturated fats and starch. In detail, at the time of data collection, the Italian diet was characterized by one of the highest intakes of starch among Western countries, with the main sources of starch—associated also with RCC risk in our dataset [14]—being white bread and pasta [25,26]. Similarly, the main source of sodium from foods (i.e., no added salt) in the Italian population was bread, which was also consumed by the largest part of the population [26], and cereal and cereal products in general, according to a national survey conducted in Italy at the beginning of the current study [27]. In addition, the use of olive and seed oils in recipes and as a dressing for salad or other raw and cooked vegetables was highly prevalent, and vegetable cooking oils and dressings are the major sources of vitamin E, mono and polyunsaturated fatty acids, in particular linoleic and linolenic fatty acids [25,26]. Finally, the Italian population at the time of data collection showed a high consumption of fruit and vegetables, which are the major sources of vitamin C, vitamin E, beta-carotene, potassium, soluble carbohydrates, and fiber [25,26]. The combination of the mentioned nutrients within each of the identified DPs shown in Table 2 fully captures the main characteristics of the Italian diet at the time of data collection.

The analysis of correlation coefficients in Table 3 further confirmed the previous interpretation considering a set of relevant food groups in the Italian diet. In detail, bread and red meat, as well as pasta and rice, characterized the “Starch-rich” DP; unspecified seed and olive oils, as well as red meat, were correlated with the “Cooking oils and dressings” DP; fruit and vegetables well described the “Vitamins and fiber” DP, with 7 of the 9 food groups including fruit and vegetables showing correlation coefficients higher than 0.25 on this DP. Table 3 also highlighted the importance of the red meat group in the Italian diet. Indeed, this food group showed non-negligible correlation coefficients with 3 of the 4 identified DPs. In detail, an additional analysis based on components of the red meat group suggested that each DP points to a different type of consumption: boiled beef is expressed in the “Animal products” DP; Bolognese pasta and schnitzel in the “Starch-rich” DP; the combination of oils with red meat that is typical of the Italian meat sauce, ragout, is expressed in the “Cooking oils and dressings” DP, together with pork chops and roast pork. If we have to justify why the red meat group has a higher correlation with the “Starch-rich” and “Cooking oils and dressings” DPs, as compared to the “Animal products” DP, we can hypothesize that the “Animal products” DP was actually more oriented towards the dairy product component (i.e., milk, eggs and cheese from Table 3), although the red meat component is still important and present.

Among possible biological mechanisms allowing starch-related foods to increase RCC risk, a high consumption of bread and pasta could elevate circulating glucose and insulin concentrations, thus promoting glucose intolerance, insulin resistance, and hyperinsulinemia. Higher levels of circulating insulin may promote cancer development by affecting insulin-like growth factor-binding proteins and by increasing IGF1 bioactivity, which has proliferative, angiogenic, antiapoptotic, and estrogen-stimulating properties [28]. In addition, high intakes of starch-rich foods may indicate a poor diet, with reduced intakes of beneficial micronutrients, inversely related to RCC risk [29]. Concerning the “Cooking oils and dressings” DP, the combination of specific fats identified in this DP could simply be an indirect marker of a diet rich in olive oil and possibly in vegetables, rather than having an active role on RCC risk. In addition, the protective effect of unsaturated fatty acids may be explained by inflammatory inhibition and alteration of free radicals production.

A direct comparison of results from the single-nutrient analysis conducted on the same dataset provided consistent results with the current one on *a posteriori* DPs. A direct association of even higher magnitude was found for starch intake, whereas an inverse association with RCC was identified for fats from vegetable sources, including vitamin E [29], linoleic and linolenic acids [30].

Factor analysis is still the most used method to identify *a posteriori* DPs in studies on diet and cancer [31,32]. Among the key advantages of factor analysis over the single-food or -nutrient approach, we mention the fact that it naturally accounts for correlations between dietary components and, as a consequence, it provides a comprehensive picture of the overall diet in a population [9]. Factor analysis is amenable to be used on continuous variables; for this reason, we chose to investigate the correlation structure among nutrients, instead of considering the potentially equivalent food groups. In addition, nutrient-based DPs may be more easily compared across different dietary sources, time-points, and populations, because they point to the key biological processes behind the single diets and do not depend on specific food groups [33,34,35]. The most important limitation of this method derives from its data-driven nature and consists in the limited generalizability of the *a posteriori* DPs to different settings, populations, and specific sub-populations [36]. This is partly due to a series of subjective decisions that researchers should take in identifying DPs, including the number and type of dietary components to be included in the analysis, the choice to carry out the procedure on controls only or on cases and controls together, the number of DPs to retain, the (possible) rotation method to use, and the interpretation of the factors in terms of DPs. To assess the potential effect of some of these choices, we performed a series of additional analyses. These checks reassured on the (so called) internal reproducibility of the identified DPs, no matter of the approach taken during factor analysis.

The present work shares strengths and limitations with any case-control study. Selection and information bias have to be mentioned. However, the similar catchment areas for cases and controls and interview set-up, as well as the (likely) limited awareness of a role of diet on RCC risk in the Italian population, reassure against the two biases. The participation rates in cases and controls were equal to 95%. In addition, we did not include in the control group subjects with diagnoses potentially associated to dietary modifications and/or having recognized risk factors for RCC. Other strengths of our study are a reproducible and valid FFQ [18,19] and the allowance for major potential confounding factors, including accurate adjustment for tobacco smoking, alcohol drinking, BMI, hypertension, and family history of kidney cancer. Like most FFQs, our tool assessed food intake with reference to the two years prior to RCC diagnosis or hospitalization. However, most RCCs are frequently asymptomatic for a long time prior to diagnosis. By design, subjects with recent changes in dietary patterns were excluded from the interview. For this reason, in the absence of major changes in food supply during the study period, we can reasonably assume that the DPs representing the two years of pre-diagnosis were conceivably reflective of longer-term DPs, and therefore the associations drawn were biologically plausible.

In conclusion, our study based on DPs from Italy suggests that intakes of starch-related foods may increase RCC risk, whereas consumption of olive and seed oils may favorably affect RCC risk.

## Figures and Tables

**Table 1:** Factorability of the correlation matrix of the original nutrients: Bartlett’s test of sphericity and measures of sampling adequacy.

Bartlett’s Test of Sphericity: *p*-Value < 0.001	
Kaiser–Meyer–Olkin Statistic—Overall Measure of Sampling Adequacy ^a^: 0.84	
Individual Measures of Sampling Adequacy:	
<0.60	Retinol
0.60–0.70	Linoleic acid, monounsaturated fatty acids
0.70–0.80	Calcium, lycopene, vitamin D, vitamin E, total fiber
0.80–0.90	animal protein, vegetable protein, cholesterol, saturated fatty acids, Other polyunsaturated fatty acids, starch, potassium, phosphorus, iron, thiamin, riboflavin, total folate, niacin, vitamin C
≥0.90	Linolenic acid, soluble carbohydrates, sodium, zinc, vitamin B6, beta-carotene equivalents

^a^ Overall and individual measures of sampling adequacy range between 0 and 1, with values > 0.60 indicating a satisfactory size.

**Table 2:** Factor loading matrix ^a^, communalities (COMM) and explained variances (VAR) for the four major dietary patterns identified by factor analysis.

Nutrient	Animal Products	Starch-Rich	Vitamins and Fiber	Cooking Oils and Dressings	COMM
Animal protein ^b^	**0.82**	0.32	-	0.34	0.90
Vegetable protein	0.15	**0.86**	0.37	0.19	0.93
Cholesterol	**0.72**	0.36	-	0.34	0.77
Saturated fatty acids	**0.71**	0.23	0.23	0.40	0.77
Monounsaturated fatty acids	0.33	0.28	0.25	0.59	0.60
Linoleic acid	0.19	0.11	0.17	**0.80**	0.72
Linolenic acid	0.34	0.11	0.18	**0.75**	0.72
Other polyunsaturated fatty acids	0.34	0.44	−0.16	0.60	0.70
Soluble carbohydrates	0.40	0.21	**0.68**	-	0.66
Starch	0.16	**0.88**	0.18	0.13	0.85
Sodium	0.44	**0.75**	0.13	-	0.78
Calcium	**0.83**	-	0.37	-	0.82
Potassium	0.46	0.43	**0.65**	0.26	0.90
Phosphorus	**0.78**	0.42	0.33	0.23	0.94
Iron	0.43	0.53	0.35	0.33	0.70
Zinc	**0.64**	0.58	0.22	0.37	0.93
Thiamin (vitamin B1)	0.54	0.52	0.49	0.25	0.86
Riboflavin (vitamin B2)	**0.80**	0.19	0.44	0.14	0.89
Vitamin B6	0.51	0.51	0.48	0.36	0.88
Total folate	0.42	0.42	**0.63**	0.28	0.83
Niacin	0.41	0.61	0.23	0.43	0.77
Vitamin C	0.11	-	**0.82**	-	0.69
Retinol	0.41	-	-	0.16	0.20
Beta-carotene equivalents	0.10	-	**0.67**	0.30	0.55
Lycopene	-	0.39	0.32	0.36	0.39
Vitamin D	0.35	0.39	−0.14	0.35	0.42
Vitamin E	0.19	0.18	0.44	**0.81**	0.92
Total fiber (Englyst)	-	0.44	**0.76**	0.12	0.79
**Proportion of VAR explained (%)**	54.42	8.03	6.19	5.88	
**Cumulative VAR explained (%)**	54.42	62.45	68.64	74.52	

VAR: variance. ^a^ Estimates from a principal component factor analysis performed on 28 nutrients. The magnitude of each loading measures the importance of the corresponding nutrient to the factor. ^b^ Loadings greater or equal to 0.63 defined dominant nutrients for each factor, and were shown in bold typeface; loadings smaller than 0.1 were suppressed.

**Table 3:** Spearman rank correlation coefficients between continuous factor scores derived from factor analysis on nutrient intakes and weekly number of portions for 29 selected food groups defined on the same data.

Food Group	Animal Products	Starch-Rich	Vitamins and Fiber	Cooking Oils and Dressings
Milk ^a^	**0.48**	−0.19	**0.25**	−0.21
Coffee ^a^	-	0.16	-	-
Tea and decaffeinated coffee	-	-	0.16	-
Bread	-	**0.74**	-	-
Pasta and rice	-	0.23	0.14	0.14
Soup	0.10	-	0.12	-
Eggs	**0.30**	-	-	0.21
White meat	0.17	-	-	0.15
Red meat	**0.28**	**0.34**	−0.14	**0.38**
Liver	**0.29**	-	-	0.14
Processed meat	0.23	0.23	-	0.11
Fish	0.10	0.23	−0.11	0.21
Cheese	**0.52**	-	-	-
Potatoes	0.15	0.13	-	0.21
Pulses	-	0.13	**0.27**	-
Leafy vegetables	-	−0.11	**0.28**	0.21
Fruiting vegetables	-	-	**0.38**	0.18
Root vegetables	-	-	**0.30**	0.12
Cruciferous vegetables	-	-	0.17	-
Other vegetables	0.10	-	**0.32**	0.18
Citrus fruit	-	-	**0.46**	-
Other fruit	-	-	**0.61**	-
Soft drinks and fruit juice	0.10	-	-	-
Desserts	**0.29**	0.17	0.14	-
Sugar and candies	0.22	0.16	-	-
Butter and margarine	0.20	-	0.12	0.24
Specified seed oils	-	-	-	0.18
Unspecified seed oils	0.16	0.13	−0.19	**0.46**
Olive oil	-	0.22	0.21	**0.32**

^a^ Correlations greater or equal to 0.25 (in absolute value) were shown in bold typeface; correlations smaller than 0.1 (in absolute value) were suppressed.

**Table 4:** Baseline characteristics of the study participants according to quartiles of dietary patterns.

Dietary Pattern, Quartile	Number of Cases/Controls	Ever Alcohol Drinking ≥ 21 Drinks/ Week (%)	Current Tobacco Smoking (%)	Hypertension (%)	Family History of Kidney Cancer (%)	Body Mass Index (kg/m^2^) ^a^	Energy Intake (kcal/day) ^a^
Animal products							
1 (low)	193/384	22.5	29.1	27.4	2.1	26.2	1989.6
2	185/383	27.1	30.5	31.7	1.2	26.2	2145.0
3	222/383	31.9	31.2	32.6	0.5	26.0	2351.3
4 (high)	167/384	37.2	31.6	23.2	0.7	26.2	2839.7
*p*-value ^b^		<0.0001	0.343	0.204	0.014	0.996	<0.0001
Starch-rich							
1 (low)	159/384	18.4	22.1	31.5	1.1	26.0	1844.8
2	185/383	28.0	30.1	31.0	1.6	26.0	2057.9
3	216/384	30.3	31.0	29.5	0.8	26.2	2380.4
4 (high)	207/383	40.9	38.5	23.6	1.0	26.4	2936.6
*p*-value ^b^		<0.0001	<0.0001	0.003	0.597	0.040	<0.0001
Vitamins and fiber							
1 (low)	205/384	30.4	35.1	31.1	1.0	26.1	2084.7
2	189/383	29.7	31.6	29.0	1.1	26.1	2233.8
3	186/384	29.1	27.5	30.4	1.4	26.6	2313.5
4 (high)	187/383	29.3	27.9	24.7	1.1	26.0	2584.9
*p*-value ^b^		0.647	0.003	0.036	0.815	0.924	<0.0001
Cooking oils and dressings^c^							
1 (low)	246/384	26.2	31.8	32.4	0.8	25.4	2103.5
2	188/383	27.3	30.0	26.8	1.2	26.0	2111.2
3	176/383	27.6	29.9	29.0	0.9	26.6	2270.0
4 (high)	157/384	38.3	30.7	26.6	1.7	26.8	2821.6
*p*-value ^b^		<0.0001	0.678	0.064	0.249	<0.0001	<0.0001

^a^ Median within each quartile category of the dietary patterns. ^b^ Trend across quartiles according to Cochran-Armitage test for categorical variables.

**Table 5:** Odds Ratios (ORs) ^a^ of renal cell carcinoma and corresponding 95% Confidence Intervals (CIs) on quartiles of factor scores from a principal component factor analysis.

Dietary Pattern	Quartile Category, OR (95% CI)				P_trend_^c^
	I ^b^	II	III	IV	
Animal products	1	0.92 (0.72–1.20)	1.13 (0.88–1.45)	0.87 (0.67–1.14)	0.629
Starch-rich	1	1.18 (0.90–1.54)	1.45 (1.11–1.89)	1.38 (1.04–1.82)	0.018
Vitamins and fiber	1	0.97 (0.76–1.26)	0.93 (0.72–1.20)	1.02 (0.79–1.32)	0.935
Cooking oils and dressings	1	0.74 (0.58–0.95)	0.71 (0.55–0.91)	0.61 (0.47–0.80)	<0.001

^a^ Estimates from conditional logistic regression model, conditioned on center, sex, and age and adjusted for period of interview, education, body mass index, tobacco smoking, alcohol drinking, family history of kidney cancer and hypertension. Results refer to the composite model including all the four factors together. ^b^ Reference category. ^c^ *p*-value for linear trend.

**Table 6:** Odds ratios (ORs) ^a^ of renal cell carcinoma and corresponding 95% confidence intervals (CIs) on quartiles of factor scores in strata of body mass index (BMI), hypertension, and education.

Dietary Pattern		Quartile Category, OR (95% CI)				P_strata_^c^
		I ^b^	II	III	IV	
Animal products	BMI < 30	1	0.89 (0.67–1.19)	1.16 (0.88–1.53)	0.88 (0.66–1.17)	
	BMI ≥ 30	1	1.21 (0.62–2.38)	1.15 (0.58–2.25)	0.81 (0.39–1.65)	0.769
	No hypertension	1	1.01 (0.74–1.39)	1.22 (0.90–1.67)	1.02 (0.74–1.39)	
	Hypertension	1	0.77 (0.48–1.23)	0.99 (0.63–1.55)	0.65 (0.38–1.08)	0.533
	Education <11 years	1	1.02 (0.76–1.37)	1.32 (0.99–1.76)	1.02 (0.75–1.39)	
	Education ≥11 years	1	0.67 (0.38–1.18)	0.77 (0.45–1.32)	0.51 (0.29–0.90)	0.151
Starch-rich	BMI < 30	1	1.20 (0.89–1.61)	1.45 (1.08–1.94)	1.25 (0.92–1.70)	
	BMI ≥ 30	1	1.07 (0.53–2.17)	1.38 (0.69–2.76)	2.35 (1.16–4.78)	0.331
	No hypertension	1	1.08 (0.77–1.50)	1.24 (0.89–1.72)	1.25 (0.89–1.74)	
	Hypertension	1	1.35 (0.84–2.16)	1.90 (1.18–3.06)	1.62 (0.97–2.71)	0.680
	Education <11 years	1	1.24 (0.92–1.67)	1.37 (1.01–1.85)	1.43 (1.04–1.96)	
	Education ≥11 years	1	1.00 (0.54–1.85)	1.81 (1.00–3.29)	1.44 (0.78–2.66)	0.528
Vitamins and fiber	BMI < 30	1	0.85 (0.65–1.13)	0.83 (0.63–1.10)	0.95 (0.72–1.26)	
	BMI ≥ 30	1	2.41 (1.20–4.83)	1.89 (0.93–3.81)	1.75 (0.83–3.67)	0.052
	No Hypertension	1	1.02 (0.75–1.40)	0.97 (0.71–1.34)	1.07 (0.78–1.46)	
	Hypertension	1	0.91 (0.58–1.44)	0.91 (0.58–1.42)	0.85 (0.53–1.36)	0.760
	Education <11 years	1	1.00 (0.75–1.33)	0.96 (0.71–1.28)	1.12 (0.83–1.50)	
	Education ≥11 years	1	0.86 (0.49–1.51)	0.82 (0.47–1.43)	0.73 (0.42–1.26)	0.658
Cooking oils and dressings	BMI < 30	1	0.75 (0.57–0.98)	0.68 (0.52–0.90)	0.61 (0.46–0.81)	
	BMI ≥ 30	1	0.66 (0.33–1.35)	0.75 (0.37–1.53)	0.62 (0.31–1.23)	0.878
	No hypertension	1	0.67 (0.50–0.90)	0.59 (0.43–0.81)	0.56 (0.40–0.76)	
	Hypertension	1	0.90 (0.56–1.42)	0.94 (0.60–1.47)	0.70 (0.43–1.14)	0.128
	Education <11 years	1	0.69 (0.52–0.92)	0.70 (0.52–0.93)	0.61 (0.46–0.82)	
	Education ≥11 years	1	0.90 (0.54–1.52)	0.77 (0.45–1.30)	0.60 (0.33–1.11)	0.618

^a^ Estimates from conditional logistic regression model, conditioned on center, sex, and age and adjusted for period of interview, education, body mass index, tobacco smoking, alcohol drinking, family history of kidney cancer and hypertension when not considered as a stratification variable. Results refer to the composite model including all the four factors simultaneously. ^b^ Reference category. ^c^ *p*-value for heterogeneity.

**Table 7:** Odds ratios (ORs) ^a^ of renal cell carcinoma and corresponding 95% confidence intervals (CIs) on quartiles of factor scores in strata of tobacco smoking and alcohol drinking.

Dietary Pattern		Quartile Category, OR (95% CI)				P_strata_^c^
		I ^b^	II	III	IV	
**Animal products**	Never smoker	1	0.93 (0.62–1.41)	1.25 (0.84–1.86)	0.81 (0.52–1.24)	
	Former smoker	1	0.97 (0.60–1.58)	0.92 (0.56–1.50)	0.63 (0.38–1.06)	
	Current smoker <20 c/d	1	0.44 (0.20–0.97)	1.32 (0.66–2.63)	0.93 (0.46.1.88)	
	Current smoker ≥20 c/d	1	1.66 (0.82–3.37)	1.78 (0.87–3.67)	1.96 (0.90–4.28)	0.013
	Alcohol drinker <21 d/w	1	0.97 (0.72–1.30)	1.12 (0.84–1.50)	0.86 (0.62–1.18)	
	Alcohol drinker ≥21 d/w	1	0.81 (0.47–1.39)	1.23 (0.74–2.05)	0.92 (0.55–1.54)	0.803
Starch-rich	Never smoker	1	1.12 (0.76–1.66)	1.21 (0.80–1.81)	1.24 (0.79–1.94)	
	Former smoker	1	1.15 (0.65–2.02)	1.59 (0.93–2.73)	1.69 (0.98–2.94)	
	Current smoker <20 c/d	1	1.50 (0.69–3.28)	2.48 (1.12–5.46)	1.83 (0.82–4.09)	
	Current smoker ≥20 c/d	1	1.55 (0.66–3.62)	1.63 (0.71–3.73)	1.18 (0.53–2.64)	0.178
	Alcohol drinker <21 d/w	1	1.04 (0.77–1.41)	1.37 (1.01–1.85)	1.24 (0.89–1.73)	
	Alcohol drinker ≥21 d/w	1	1.73 (0.94–3.18)	1.89 (1.03–3.45)	1.79 (1.00–3.24)	0.391
Vitamins and fiber	Never smoker	1	1.39 (0.91–2.13)	1.39 (0.92–2.12)	1.48 (0.96–2.27)	
	Former smoker	1	0.84 (0.52–1.37)	0.74 (0.45–1.20)	0.68 (0.41–1.12)	
	Current smoker <20 c/d	1	0.68 (0.35–1.32)	0.46 (0.22–0.97)	0.85 (0.43–1.69)	
	Current smoker ≥20 c/d	1	0.76 (0.38–1.50)	1.43 (0.72–2.83)	1.24 (0.62–2.49)	0.019
	Alcohol drinker <21 d/w	1	1.00 (0.74–1.35)	0.88 (0.65–1.19)	0.94 (0.69–1.28)	
	Alcohol drinker ≥21 d/w	1	0.82 (0.50–1.33)	0.95 (0.59–1.55)	1.08 (0.67–1.75)	0.582
Cooking oils and dressings	Never smoker	1	0.72 (0.48–1.06)	0.63 (0.42–0.94)	0.60 (0.39–0.93)	
	Former smoker	1	0.66 (0.40–1.08)	0.59 (0.35–0.99)	0.49 (0.29–0.81)	
	Current smoker <20 c/d	1	1.15 (0.59–2.23)	0.68 (0.34–1.34)	0.87 (0.42–1.83)	
	Current smoker ≥20 c/d	1	0.60 (0.30–1.21)	0.92 (0.44–1.93)	0.60 (0.30–1.18)	0.421
	Alcohol drinker <21 d/w	1	0.79 (0.59–1.06)	0.76 (0.57–1.03)	0.69 (0.50–0.94)	
	Alcohol drinker ≥21 d/w	1	0.56 (0.34–0.92)	0.50 (0.30–0.82)	0.44 (0.28–0.71)	0.463

c/d: cigarettes/day; d/w: drinks/week. ^a^ Estimates from conditional logistic regression model, conditioned on center, sex, and age and adjusted for period of interview, education, body mass index, tobacco smoking, alcohol drinking, family history of kidney cancer and hypertension, when not considered as a stratification variable. Results refer to the composite model including all the four factors simultaneously. ^b^ Reference category. ^c^ *p*-value for heterogeneity.
